# Barriers to managing and delivery of care to war-injured survivors or patients with non-communicable disease: a qualitative study of Palestinian patients’ and policy-makers’ perspectives

**DOI:** 10.1186/s12913-020-05302-6

**Published:** 2020-05-11

**Authors:** Marwan Mosleh, Yousef Al Jeesh, Koustuv Dalal, Charli Eriksson, Heidi Carlerby, Eija Viitasara

**Affiliations:** 1 Ministry of Health, Gaza, Palestine; 2 Department of Health Sciences (HLV), Public Health Science, Mid Sweden University, Sundsvall, Sweden; 3 International Public Health Medicine, Islamic University, Gaza, Palestine; 4 Higher School of Public Health, Al Farabi Kazakh National University, Almaty, Kazakhstan; 5 Department of Public Health Sciences, Stockholm University, Stockholm, Sweden

**Keywords:** Barriers, NCD, War injured survivors, Policy makers, Healthcare, Palestine

## Abstract

**Background:**

Improving access to optimal quality of care is a core priority and ambitious health policy goal in spite of impediments, threats and challenges in Palestine. Understanding the factors that may impede quality of care is essential in developing an effective healthcare intervention for patient with non-communicable disease (NCD) or war-injured survivors.

**Methods:**

Qualitative interviews were performed using a purposive sampling strategy of 18 political-key informants, 10 patients with NCD and 7 war-injured survivors from different health facilities in Gaza Strip. A semi-structured interview guide was developed for data collection. The interviews were audio recorded and transcribed verbatim. Important field notes of the individual interviews were also reported. Thematic-driven analytic approach was used to identify key themes and patterns.

**Results:**

From the policy maker’s perspective, the following important barriers to accessing optimal healthcare for patients with NCD or war-injured survivors’ treatment were identified; 1) organizational/structural 2) availability 3) communication 4) personnel/lack of staff 5) financial and political barriers. Patient with NCD or war-injury had similar experiences of barriers as the policy makers. In addition, they also identified socioeconomic, physical and psychological barriers for accessing optimal healthcare and treatment.

**Conclusions:**

The main perceived barriers explored through this study will be very interesting and useful if they are considered seriously and handled carefully, in order to ensure efficient, productive, cost-effective intervention and delivery of a high-standard quality of care and better disease management**.**

## Background

Globally, NCDs, e.g. cardiovascular disease, cancers, chronic respiratory diseases and diabetes are a growing health concern [1, 2], and projected to be the leading cause of mortality and disability by 2030 [3, 4]. In 2005, the World Health Organization (WHO) announced a new global goal to tackle the increasing trend of mortality of NCDs by 2% annually. Prevention and managing of NCDs are important strategies to achieving this goal [5]. General practice could play a significant role in delivering high-quality care for a range of high-prevalence NCD, and ensuring continuity of care for co-morbidity [6]. The management of NCD will be one of the most significant health challenges of the modern century [7], although most cases could be better prevented or managed [8]. The main goal of NCD prevention and management policy is to prevent disease development and improve the quality of population health [9]. The rapid increase in the trend of NCD represents a big challenge to healthcare and disease management globally, especially in low and middle-income countries (LMICs) like Palestine [10–12].

War-related injury also represents a critical public health concern globally. It receives increasing attention in health research. Such injury is a tremendous burden for the healthcare sector and economy, especially in LMICs [13–15]. The availability of adequate healthcare and resources will efficiently affect the outcome of surgical interventions for war-injured survivors [16]. Most importantly, prehospital trauma care is potentially life-saving for war-injured survivors due to a shorter evacuation time [17–19], while the prolonged evacuation time may be associated with higher mortality [20]. War-related injury cases ideally are managed by experienced hospital surgeons who have sufficient skills of surgical management [21]. Access to high-quality health care remains a significant health problem particularly for patients who need a longer course of treatment [22]. However, the provision of adequate health care specially during economic collapse in the country is an important health concern to ensure optimal care [23–25].

In developing countries such as Arab world, access to equitable, comprehensive and quality health services represents a challenge to health care systems that are disproportionately being affected by many barriers to access to proper and affordable healthcare [26]. In order to reduce the burden of these health issues, reorientation of healthcare is a core element in the health system. Many countries have adopted a new policy to provide of health services for all people and to ensure their availability, accessibility and patient-centered quality [27].

In the Palestinian territory, the management of patients with NCDs and war-injured survivors, as well as provision of care pose a challenge to the health system during and after emergency, especially in the Gaza Strip [14, 28], where, the health care system is fragmented and continues to face many challenges. Many health facilities are severely overstretched due to conflicts and frequent wars on Gaza [29]. The health care systems in Gaza, and life-saving medical resources already depleted and fragile [30, 31].

The care for NCD conditions and war-injured survivor remains the priority for the healthcare system, despite limited resources and numerous barriers to access effective healthcare, which may result in poorer outcomes [32].

Understanding the factors and barriers that may impede quality of care is essential in developing effective health care interventions. Unfortunately, war-injured survivors care and NCDs management have been largely neglected and received little attention in health services research. Therefore, it is necessary to investigate the main perceived barriers to effective management for patients with NCD and war-injured survivors in Palestine. The aim of the study is to explore patients’ experience and policy makers’ views on the barriers to reach the quality of health care. It would be step forward and significant information to improve health services for patients with NCD and war-injured survivors in the Palestinian health system.

## Methods

### Study design

This qualitative exploratory study was conducted between December 2018 and March 2019 in the Ministry of Health (MOH) facilities and hospitals in Gaza Strip to explore the perceived barriers associated with delivery of care as well as NCD and war-injured survivors management. A qualitative method is the most common approach and widely involved in health research and is best recommended as to give insight rather than measurement, as it is exploratory in nature [33, 34]. Qualitative research is increasingly receiving attention to understand challenges in health care settings [35].

### Informants and data collection

A total of 35 informants were purposively selected (Table 1). Sex, age, location and socio-economic background were considered to ensure their representativeness in the study. The sampling was completed when thematic saturation was achieved, meaning that sufficient data were collected on the topic and no new themes were emerging from interviews. Each hospital or MOH facility was contacted asked to give us the contact details of a key informants who think they had adequate information and were willing to be involved in our study.

**Table 1:** Categories of informants for in-depth interviews

Informants	In-depth interviews
Patients	
Patients with NCD	10
War-injured survivors	7
Policy makers	18
Total	35

The policy makers were recruited from different positions, specialties and administrative levels in the public hospitals and MOH departments to ensure representativeness of the studied populations. They were invited as “information-rich informants” with sufficient knowledge and experience on barriers to providing quality care to the patients and recommendations to improve the quality of health care and disease management. They included 18 political informants ages between 40 and 58 years and had work experience of at least 15 years. The majority of political informants were males. They were invited based on the relevance of their position within medical, surgical and administrative fields at MOH facilities. They included general managers, medical and surgical managers, directors of nursing, head of health administration, and director of NCD division who were involved in health policy and planning at MOH.

The patients included 10 patients with NCD and 7 war-injured survivors, their ages ranged from 30 to 59 years and most of them were males. The patients with NCD were recruited from the main three public hospitals in Gaza Strip governorates. The sample included those who had cardiovascular disease (heart disease and hypertension), chronic kidney disease (e.g. renal failure), cancer, diabetes mellitus, respiratory disease, while war-injured survivors were guided and recommended by MOH with assistance from Assalama Society for Wounded and Disabled (ACSWD). All participated NCD patients or war-injured survivors has been receiving care for at least 3 years, meaning that they had adequate experience with barriers and troubles regarding their case management. The study participants received no reward or compensation in return for their participation, their involvement was voluntary and they were eager and willing to participate in the study.

The process of data collection was conducted by the principal investigator and two trained staff through face-to-face semi-structured interviews, using a special discussion guide developed by the authors based on earlier research and literature (Additional file 1). Two separate interview guides were used, one for patients and one for political-key informants. The trained staff engaged in three-day training session by the investigator before they conducted interviews, to ensure that they fully understand the aims of the study and questions of the guide. The research staff was also trained on how to deal with any sensitive issues or any indications of distress with proper response and to avoid irrelevant or complicated probing.

Before conducting the study, the questions were reviewed, refined and improved by the health experts. The interview guides were also pretested among small sample of patients and managers in one of the hospitals not considered in the study to standardize the guide. Based on informant’s request, all interviews were held in their respective language (Arabic), then translated back to English by expert fluent speakers. Each interview with political-key informants and chronically ill was conducted and moderated privately in a calm and comfortable environment in the targeted health facilities, whereas interviews with war-injured survivors were performed at ACSWD as preferred by the informants. The interviews focused on a better understanding of the key barriers and troubles in managing NCD and war-injured survivors in the Palestinian health system.

The researcher and assistance staff visited each hospital to gather relevant data. Each interview was audio-recorded, and important field notes were made. Each interview lasted 30–40 min. In the beginning of each interview, the interviewees were warmly welcomed, introduced themselves, and a short background of study aims was provided by the interviewer. The interviews started with a simple question and going on in-depth gradually, leading the discussion on the issue of the main perceived important barriers for accessing healthcare, effective NCD and war-injured survivors management issues.

#### Data analysis

Before proceeding with qualitative data analysis, person identity was deleted in order to maintain people’s privacy and anonymity. After transcriptions of interviews and handwritten notes, the data were checked and validated by peer reviewers. The data were analyzed using thematic-driven analysis method. We adopt Clarke and Braun framework of doing thematic analysis which includes six phases of analysis to identify patterns and themes relevant to research questions and elaborating data in-depth. These phases are: familiarization with data, generating initial codes, searching for themes among relevant codes, reviewing themes, defining themes, and producing the final report [33, 36, 37].

In the first step of analysis, the transcripts were read several times until becoming familiar with the data. Then, the transcripts of the data were initially coded by the principal investigator who moderated the interview sessions, and then checked by the other researchers. From initial coding we searched for the links among themes and identified key concepts. Debriefing sessions and follow-up meeting were performed between the principal investigator, research assistant and second author to discuss main themes and patterns that were not adequately obvious in the transcriptions.

The emerged codes explored in the first step of reading were matched to suitable themes, then the remaining themes were very simple and clear as the researchers became familiarized with the themes after frequent readings. The emerging themes were checked and reviewed critically and after that, the themes were defined. The analysis concluded with interpretation and writing of report. The results are reported separately for policy makers (PM) and patients (War-injured S and NCD P), and quotations were used to illuminate the results.

Trustworthiness of the study.

In order to ensure the trustworthiness and to reduce the influence of researcher, four basic criteria were applied during all stages of the study including: credibility, transferability, dependability and confirmability [38]. The credibility was achieved during prolonged engagement in the field, triangulation by using more than one data source, frequent debriefing session, peer review and member check. We used a purposive sampling form different health settings of Gaza governorates and detailed description of informants and clearly described the context to ensure transferability. Dependability and confirmability were maintained by audit trail and frequent discussion with authors and external researchers.

### Ethical issues

This study was performed according to the regulations and principles of Helsinki Declaration Ethics. This study was approved by the Palestinian Health Research Council (PHRC) for Ethics Approval (Ref. No. PHRC/HC/234/17**)**. The study was also approved, permitted and facilitated by the MOH to undertake the study at public health facilities. As human subjects are involved in our study, a written informed consent was obtained from participants. Information and aim of the study were explained to participants and they were informed that their involvement in the study is voluntary. The privacy and anonymity were maintained, and any personal information and identity were removed.

## Results

Data saturation was reached after face-to-face interviews with 18 policy makers and 17 patients exploring their perspectives and experiences regarding barriers to accessing quality of care and optimal management in the Palestinian healthcare system. We identified five themes for the policy makers (Table 2), and four themes for the patients (Table 3).

**Table 2:** Main themes and sub-themes identified/explored from interviews with policy makers

Themes	Sub-themes
**Organizational/structural barriers**	-Poor logistics and hospital infrastructure. -Lack of clear universal policy. -Higher patients load. -Unproductive triage in emergency department (sorting out system is unclear), unavailable in all public hospitals -Lack of specialized units (e.g. kidney transplantation unit). -Low health care providers motivation.
**Availability related barriers**	-Insufficient guidelines and insurance system. -Shortage of experts in sub and micro-specialties (e.g. vascular surgery & neurosurgery, cervical trauma, emergency medicine, intensive care capacity) -Interrupted drug supply and lack of medical disposables. -Lack of training opportunities -Insufficient rehabilitation services and poor referral system
**Personal barriers**	-Low staff motivation -Poor working environment/circumstances -Poor knowledge and experience to use the guidelines.
**Financial and political barriers**	-Lack funding to ministry. -Lack of political commitment by authorities. -Political division between the two Palestinian entities.
**Communication barriers**	-Weak communication among health care providers. -Insufficient interaction between health workers and patients. -Suboptimal task-sharing between MOH and stakeholders.

**Table 3:** Main themes and sub-themes identified/explored from interviews with patients

Themes	Sub-themes
**Organization barriers/health system barriers**	-Weaknesses of the referral mechanism. - High war-injured survivors’ volume in emergency unit. -Delay of care delivered by physicians/surgeons.
**Availability, financial and socioeconomic barriers**	-Insufficient staff in medical and surgical units -Lack of expertise in microsurgery (vascular and neurosurgery) and kidney disease. - Unaffordability. - Poor economic conditions and diet regimen.
**Physical and psychological barriers**	-Limitation of body movement and feeling lack of energy. -Severity of war-injury, NCD and its complications. -Poor self-care and help-seeking. -Poor psychological condition.
**Communication barriers**	-Poor clear communication system with health care provider. -Insufficient patients-provider interaction and responsiveness

### I-perspectives of policy makers

Five themes were derived from the descriptive thematic analysis:1) Organizational/structural barriers; 2) Availability related barriers; 3) Personal barriers; 4) Financial and political barriers; 5) Communication barriers. The themes reflected the perceived barriers about quality of care and NCD and war-injured survivors management from perspective of policy makers (Table 2).

### Organizational/structural barriers

The policy makers identified several barriers which are perceived as the main determinant for accessing quality of care in the health system and may result in suboptimal health outcome.

The informants stressed that lack of adequate systems and infrastructure as well as absence of a clear universal policy were significant barriers in the Palestinian health system. The number of beds (1.3 per 1000 people in Gaza Strip) and hospital capacity are substandard and could not meet the expectation of patients. In fact, the optimal health care cannot sufficiently and timely be achieved in the healthcare facilities with poor infrastructure and logistics but also due to absence of clear policy. The informants said that the triage is unproductive in all public hospitals and unavailable in some hospitals due to unclear health policy in this regard. They also stated the absence of standard triage classification or assessment system to sort cases according to priorities.

They also described higher patients load, long waiting time, and overcrowding of attendants and visitors specially in emergency and surgical wards, which impede the delivery of immediate care to patients, a significant barrier to improve health care in the Palestinian healthcare system.

*“health facilities are confronting a big challenges and obstacles such as; poor of basic*

*hospitals infrastructure, frequent power outage, and poor logistics and maintenance”* (PM).

*“there are several problems for quality of care and management such as; a non-integrated*

*and fragmentation of health system and unclear universal policy”* (PM).

*“there is absence of effective triage at public hospitals, and some areas are lacking”* (PM).

### Availability-related barriers

The informants said that there are no national unified protocols or updated clinical guidelines for management of diseases, and the management depends on the health providers’ perception and experience.

*“MOH has no recent guidelines. On the year 2000 by help of the World Bank, they*

*developed protocols for management of hypertension, diabetes and renal diseases”* (PM).

All informants said that the inadequacy of human, medical and financial resources are important barriers facing the health system, affecting the quality of care and management process. These barriers include: shortage of surgical and medical medicines and interrupted drug supply, lack of experts in sub and micro-specialties like vascular and neurosurgery, cervical trauma, emergency medicine and lack intensive care capacity, lack of training opportunities and shortage of rehabilitation services for patients with NCD or war-injured survivors. These barriers contribute considerably to unpleasant health outcome and delay of care.

*“lack of essential resources, medications and medical disposables and interrupted drug*

*supply because of the continuity of siege imposed by Israeli forces, and lack of experts in different aspects of diseases especially in micro and sub-specialties are perceived to*

*be important barriers to quality of care”* (PM).

The informants expressed the poor referral system is a major barrier to necessary quality of care due to a chronic imposed of blockade and closure on Gaza strip, causing restriction of movement and referral of critical cases abroad. Unfortunately, this continuous scenario frequently led to delay of recovery and resulted in death while waiting for referral.

*"I think. poor and complicated referral system is a major constraint to quality of care especially for*

*those who had complicated condition like sustained war injury during wars or conflicts" (*(PM).

### Personal barriers

The informants believed that there were many personal barriers affecting the quality of care and case management, which include low staff motivation due to lack of incentives and salaries, lack of knowledge and experience, lack of interest, poor supervision and monitoring to use practice guidelines.

*“ … there are many factors such as lack of interest, lack of awareness, lack of*

*knowledge and experience to follow guideline, lack of priority, and lack of supervision*

*and follow-up”* (PM).

*"lack of incentives and wages are most important barriers among staff"* (PM).

### Financial and political barriers

The informants expressed several concerns reported on financial issue barriers as a fundamental determinant for accessing suitable health care. The informants stated that inadequate health insurance coverage was perceived a key barrier to effective care in the public hospitals in Gaza strip. It is a matter of fact that, without health insurance coverage, primary, secondary or even rehabilitation care could not sufficiently and timely be accessed. If such care is not timely and sufficiently available at public health facilities, the care at private facilities are often not covered by health insurance and costs are very high for most people. However, payment for care at private facility is required and it is costly and unaffordable for most people especially those with low socioeconomic status.

Another important challenge is lack of funding to the ministry for the provision of basic resources in health facilities, which affects the delivery of optimal quality of care to meet the expectations and aspirations of people.

*“I think there are many constraints that impede the shared-care and cooperation*

*between health sectors in Gaza strip such as lack of health insurance coverage*

*and lack of financial support”* (PM).

The policy makers described that the Gaza strip is a setting of a protracted political and socioeconomic crises which affects directly or indirectly the quality of care and healthcare system in general. There are numerous political barriers explored by the policy makers which may impede the quality of care in the health facilities including: lack of political commitment by authorities as well as political division between the main two Palestinian entities; the Gaza strip and West bank.

*“there is political division between the two Palestinian territories”* (PM).

*"in addition, division of health system and lack of unified decision and political*

*commitment by the authorities”* (PM).

### Communication barriers

Effective communication was perceived as a very crucial issue for optimal care and better case management. However, there are many important perceived barriers which constrain this process in the Palestinian health facilities including: absence of adopted clear official communication system between health care providers, lack of communication skills and required facilities, weak interaction between health workers and patients, and suboptimal shared-care between MOH and relevant stakeholders.

*“I believe that lack of productive communication and interactions between patient-health*

*professionals, and lack coordination among various health facilities were more likely to be*

*perceived as the essential problems to effective care and management”* (PM).

### II- patients’ experiences

Four themes were derived from the descriptive thematic analysis with patients: 1) Organizational/health system barriers; 2) Availability, financial and socioeconomic barriers;3) Physical and psychological barriers; 4) Communication barriers (Table 3).

### Organization barriers/health system barriers

The patients expressed a concern regarding organization and administration issue in hospitals, as well as bored routine and bureaucratic work. Additionally, overcrowding of attendants and high patient volume in emergency ward was also perceived the commonest barriers to receive good care from healthcare providers in public hospitals.

*"overcrowding of cases and attendants, long-waiting and appointment time and lack*

*of organizing and administration in governmental health facilities"* (War-injured S).

Furthermore, delay of physicians and poor adherence to appointments are common in public hospitals, which make patients wait for a long time to receive their care and follow-up.

*“I experienced some problems such as neglecting from care providers to patient inquiries,*

*bored routine and bureaucratic, delay of treating doctor and long-waiting time*

*and more concerns”* (War-injured S).

Another important concern is the weaknesses of referral system facing the patients when the adequate health services are unavailable at public hospitals, since the referrals between public hospitals and other local health facilities as well as abroad were common. However, there were restrictions and delays to get permission from concerned authorities as well as poor coordination with the referral health facilities.

*"I faced an important problem regarding my referral abroad to continue my*

*treatment, since I waited for more than 3 years for my referral abroad but I didn’t get*

*it till now, incredible!”* (War-injured S).

### Availability, financial and socioeconomic barriers

Patients had negative experiences regarding their condition management related to diverse of barriers. Most patients experienced many barriers in accessing quality of care, which include: lack of necessary resources such as shortage of medicines (e.g. cardiac drugs, renal failure drugs, anticoagulants), lack of expertise and teamwork in kidney disease and important micro-specialties (e.g. vascular and neurosurgery), lack of medical instruments and equipment (e.g. laparoscopes, surgical tools and instruments and patient monitors) and medical imaging machines (e.g. MRI and CT scan). Patients mentioned that they were unsatisfied with the current quality of care in public hospitals, considering that insufficient human and medical resources are an important impediment to the quality of care in the Palestinian health system.

*“the care is inadequate and unsatisfactory in general, from my experience during*

*management of my war injury, I think there were a lot of obstacles such as lack of*

*resources and facilities, lack of medicines, lack of expertise team and micro-specialties,*

*and severe shortage of medical supplies and instruments … ”* (War-injured S).

The patients complained that public hospitals frequently and repeatedly ran out of medicines and prescribed drugs for their illness. Participants said that the combination of treatments was effective when available and followed; however, for a poor people they all said that they found it difficult to pay for prescribed drugs or even going to the private health sector, since the necessary payment is mostly unaffordable to people with low socioeconomic status and also to those who are experiencing hard situation in the Gaza Strip.

Patients who were less satisfied with their “general quality of life” as well as those who had a poor “health conditions” were less likely to access healthcare services at private sectors due to lack of health insurance coverage in the system.

*"lack of affordability of medicines, and lack accessibility for good quality*

*of care due to lack of financial resources and facilities"* (NCD P).

*“the major barrier for me is the bad economic condition, since it is very*

*difficult for me to pay for treatment, transportation, medical tests and having*

*special diet regime as prescribed and recommended by physician”* (War-injured S).

### Physical and psychological barriers

Patients expressed that, without good health, it is very hard to enjoy the rest of what life has to give. That’s why getting healthcare or improving health is important and should be on of their priorities. All patients felt that their illness and war injury affected their care and management of their condition. They said that limitation of body movement and deterioration of their health due to severity of illness and complications were a major barrier to self-care and help-seeking, which resulted in poor health outcome. They explained that they no longer have good mobility and thus have a physical problem to getting appropriate health care. They always find it difficult to get out of the home and also cannot do their daily activities and self-care. Many of them complain they don’t have assistance to serve them, for example to be transported to health facilities.

*“actually, the severity of my war-related injury, complications and my complicated case in general,*

*as well as limitation of movement and energy to perform daily activities were mostly the*

*main barriers for self-care at home”* (War-injured S).

Most patients Admitted that they had psychological disorder or disturbances including anxiety and feelings of hopelessness due to the severity of their condition, and prolonged treatment as well as excessive treatment costs and economic burdens. They generally find it difficult to be in the outer world, going alone to health centers. Oftentimes, patients with psychological problems feel weak and down and strive for help to fix it. This is yet another obstacle to effectively getting health care they need.

"there are many challenges that directly affect accessing to quality of care like

movement limitation, and bad psychological condition due to seriousness of

my disease and long-stage of treatment” (NCD P).

### Communication barriers

Communication problems were reported between patients and healthcare providers, causing poor coordination of patients’ care as well as unproductive patients-healthcare providers interaction. Such perceived problems may refer to poor communication between patient and health care staff. Additionally, most patients said that they didn’t get enough details about their diagnosis and illness due to lack of interaction, poor responsiveness and misunderstanding of medical jargon.

*“I think the barriers or lack of communication is referred to absence of a clear*

*official communication channels, lack of trust and confidence, lack of response to*

*my need, and poor information about my health problem by my doctor as well as*

*medications misconception”* (NCD P).

## Discussion

Adequate access to health care is a significant and basic human right. However, insufficient access can affect population health. Health systems often struggle to deliver immediate care in Gaza Strip, especially during wartimes or conflicts. Therefore, improving quality of care and ensuring patient safety are of utmost importance to tackle mortality in low-income countries such as Palestine [39]. Where, despite the efforts to strengthen the quality of health care, it remains important concern to the health system in Gaza Strip. Therefore, this study is a unique health response to the call from the health system to explore and identify of the main barriers to access high-quality care for patients with NCD and war-injured survivors management in the Gaza strip.

This study contributes to the process of exploring new challenges and problems in addition to those that already available in the literature related to NCD and injury management service delivery at health facilities.

In this study, several important barriers were explored from the perspective of patients and key policy-makers. These barriers may directly or indirectly impede the delivery of high-quality care in the Gaza hospitals. Other studies in the region have reported some similar barriers and concerns [29, 30, 32, 40].

The quality of care perceived by the informants in this study was sub-optimal due to several barriers, which impede achievement of the ambitious standard of care. The perception of quality of care by respondents was only partly in line with WHO definition of quality of care, in which healthcare should be safe, timely, effective, efficient, equitable and people-focused [39]. In fact, ensuring a sustainability of optimal health care and medical services for Palestinians was challenging for the Palestinian health system due to these barriers, which mainly include poor hospital infrastructure, chronic and long-acting shortage of resources and logistic support, interrupted drug supplies and ongoing blockage and general closure of crossing of boarders that constrain referral of critical cases abroad due to poor supplies and facilities in Gaza Strip. These results were consistent with the findings reported by the WHO during conflict escalation in Gaza Strip in 2014 [28, 41–43]. Additionally, referral system problems were also among the important perceived barriers because of movement restriction and closure of borders, preventing access of patients and healthcare providers as well. There were ineffective referral services in the Palestinian healthcare system. The results also confirm the previous outcomes of studies conducted in the Palestinian territory. This issue is very complicated especially in a Palestinian context, where the necessary facilities and resources are lacking [44–46].

Most importantly, the division or separation of the health system due to internal Palestinian conflicts was more likely to be a fundamental barrier for improving the quality of care, causing a rapid deterioration and collapse of the healthcare system in Gaza Strip. Furthermore, poor health information system, underuse of evidence-based guidelines, lack of supervision and cooperation, sub-optimal communication and partnership, absence of enough financial incentives, and lack of training opportunities were common and identified a major concern in the system, that may result in sub-optimal quality of care for case management and unsatisfactory outcome in healthcare facilities. These facts were in line with the results drawn from an assessment of healthcare system conducted in the Occupied Palestinian Territory [47].

Among the other main obstacles in the system are poor responsiveness and inactive interaction between healthcare providers and patients due to lack of effective communication. Active inter-personal interactions and facilitation of the role of patients in decision making can produce a positive impact on patient health. Those important results and issues including organization barriers, financial barriers and emotional barrier were compatible and in support with the findings in the studies in the Arab countries [48] and Hawaii [49].

### Strength and limitation of the study

The interviews of both policy makers and patients made it possible to triangulate the results, the two perspectives gave a very similar picture of the barriers to high-quality care for patients with NCD and war-injured survivors. Another important strength is that the chosen qualitative approach facilitated the exploration of a broad variety of barriers and challenges. Th triangulation was attained throughout different sources of data by sharing experiences from multiple perspectives and wide diverse views including: chronically ill from different specialties, war injured survivors, and key political-makers from different administrative levels and positions. In addition, prolonged engagement in the field throughout long work and several visits and frequent peer reviews check was important to enhance triangulation of the data.

Despite the great importance and interesting findings of the study, there were some limitations which need to be considered. One limitation of this study is that the findings may not be transferable to other health setting, as this qualitative study is exploratory in nature and our participants were selected from specific public hospitals and the themes obtained from a narrow slice of participants.

The qualitative methods also address the phenomena in-depth, not to produce a representative finding in the statistical sense [50, 51]. However, the number of interviews were increased until saturation, where no more information was retrieved. Therefore, the variation of barriers was covered. However, the prevalence of barriers could be investigated in a study using quantitative approach.

### Implications of the study outcome

Study results have many important implications for further researches and health care, by providing significant data on the main barriers to optimal quality of care. This will be useful if properly considered and prioritized in health policy agendas and health planning. It is possible that by providing a clear description of perceived barriers, this knowledge may help to improve the access to high-quality care by tackling these barriers and facilitating management for patients with NCD and war-injured survivors in the Palestinian health facilities. The health policy must respond to many obstacles and actual strategies and action plans to design, incorporate and implement changes in order to achieve an effective healthcare.

Interestingly, the findings of this study could be a significant step and baseline for future research on the evaluation of the healthcare sector in Gaza Strip. Local knowledge is very scarce on these crucial health issues. Furthermore, the results from this study could be valuable and beneficial for health professionals in clinical practice to be aware of the problems and barriers, so that they can properly deal with them and handled the situation carefully and accurately as well.

### Perspectives of policymakers to improve the system

The policy makers suggest some area to be improved and integrated in the system in order to optimize the delivery of care. The most important issues that need to be addressed are adoption of NCD related protocols/guidelines (e.g. piloting WHO) in public hospitals, encourage policy commitment with health providers and assign responsible technical body to ensure universal implementation, and the prioritization of research in NCD related field. Furthermore, the policy makers should assure development and activation of triage in emergency departments at public hospitals, as well as improving post-operation care at hospitals. The triage of emergency at public hospitals should be well-equipped with emergency tools and instruments (e.g. emergency trolly, monitors, and medicines).

The informants advised that WHO should lead the process of implementing the national strategy of NCD. Additionally, assessment of training needs of staff (physicians, nurses, pharmacist, new graduates) as well as unified protocols are needed. Moreover, the importance of monitoring of protocols need to be emphasized during field implementation. Informants specified the need of regular meetings among stakeholders to monitor implementation and up-coming challenges. An important concern is to improve the referral mechanism and the professional link among PHC, hospitals and relevant stakeholders. The policy makers stressed that the health facilities need adequate equipment, lab kits, screening tools (scales, measuring devices) and increasing community awareness. The policy makers support the improvement of medication disposition and their storage system, and training of health staff and community members.

## Conclusions

This study provides insight into the several important barriers that the policy makers and patients perceived for effective disease management for patients with NCDs and war-injured survivors. If immediate and careful proper actions are taken towards these important barriers very interesting results will appear. These may include provision of a high-standard quality of care and improvement in productivity and effectiveness of health care in the Palestinian health facilities. A clear and comprehensive policy should be outlined and adopted that would include improving awareness and attitudes regarding these important health concerns, and how these problems can be avoidable in practice.

## Supplementary information

**Additional file 1.** Study discussion guides.

## Data Availability

Not applicable, as the data extracted through the qualitative interviews’ strategy are not publicly available.

## Abbreviations

ACSWD: Assalama Society for Wounded and Disabled
LMICs: Low- and middle-income countries
MOH: Ministry of Health
NCD: Non-communicable disease
PHRC: Palestinian Health Research Council
PM: Policy makers
WHO: World Health Organization
