# Supplementary material for: Barriers to managing and delivery of care to war-injured survivors or patients with non-communicable disease: a qualitative study of Palestinian patients’ and policy-makers’ perspectives
Source: BMC Health Serv Res. 2020 May 11;20:406. doi: 10.1186/s12913-020-05302-6 (PMC7212682; doi:10.1186/s12913-020-05302-6)
Supplement: Supplementary file 1 — Additional file 1. Study discussion guides. [file 12913_2020_5302_MOESM1_ESM.pdf]

In-depth Interview Questions Guide (English version)

Barriers to managing and delivery of care to war-injured survivors or patients with non-communicable disease: A qualitative study of Palestinian patients' and policy-makers' perspectives

I. Target participants: patients (with NCD or war injury)

**Date of interview: --**
**Place of Interview: ---**
**Time Interview started: ---------------------------------**
**Time Interview completed: ------------------------------**
**Name of moderator: ---------------------------------------**
**Name of note taker/assistant: ---------------------------**
**Code: --**

Introduction:

Thanks for participation and sharing your knowledge and treatment experience in this interview. The core subject of this research is: perceived barriers to NCDs /war injuries management in the Palestinian health facilities from your perspective as patients". ***The objective of this study:*** To explore patients and policy makers perceptions about barriers to delivery of care and management of NCDs or war injuries that could possibly help explain poor health outcome among patients with NCDs or war injured survivors, in order to contribute in improving and developing of care and clinical practice and management. To achieve this aim, we would kindly like to share us your knowledge and experiences story during your illness management.

Participants bio-data

Age: ------------- gender: ------------residence: ------------illness----------place of care---------

1.In your opinion, what have been the main barriers and challenges for accessing and using quality of care and health services for your chronic illness/war injury management?

…………………………………………………………………………………………………
…………………………………………………………………………………………………
…………………………………………………………………………………………………
…………………………………………………………………………………………………

2.What have been the main barriers for communication with your healthcare providers or care giver regarding your condition care, that you have experiences during management of your chronic illness/war injury?

…………………………………………………………………………………………………
…………………………………………………………………………………………………
…………………………………………………………………………………………………
…………………………………………………………………………………………………

3.From your treatment experience, what have been the main problems/barriers to self-management/care of your chronic illness/war injury?

What factors affecting your compliance or adherence to treatment plan, dietary regime and management instructions of your case?

…………………………………………………………………………………………………
…………………………………………………………………………………………………
…………………………………………………………………………………………………
…………………………………………………………………………………………………

4.What is your suggestion for improving and developing the quality of care and health services as well as access to suitable and timely healthcare for better disease/injury management?

…………………………………………………………………………………………………
…………………………………………………………………………………………………
…………………………………………………………………………………………………
…………………………………………………………………………………………………

5.As a patient with chronic illness/war injury, could you please explain, during wartime where you've got the care, how and by whom?

…………………………………………………………………………………………………
…………………………………………………………………………………………………
…………………………………………………………………………………………………
…………………………………………………………………………………………………

6.Finally, is there anything else that you would like to add or to share with us?

…………………………………………………………………………………………………
…………………………………………………………………………………………………
…………………………………………………………………………………………………
…………………………………………………………………………………………………

The end of patient's interview

In-depth Interview Questions Guide (English version)

Barriers to managing and delivery of care to war-injured survivors or patients with non-communicable disease: A qualitative study of Palestinian patients' and policy-makers' perspectives

II. Target participants: policy makers (from all positions and specialties)

Date of interview: --
Place of Interview: ---
Time Interview started: ---------------------------------
Time Interview completed: ------------------------------
Name of moderator: ---------------------------------------
Name of note taker/assistant: ---------------------------
Code: --

Introduction:

Thanks so much for participation and sharing your professional experience in this interview. The core focus of this discussion is: perceived barriers to NCDs/war injuries management in the Palestinian health facilities from your perspective as policy makers. ***The objective of this study:*** To explore patients and policy makers perceptions about barriers to delivery of care and management of NCDs or war injuries that could possibly help explain poor health outcome among patients with NCDs or war injured survivors, in order to contribute in improving and developing of care and clinical practice and management. To achieve this aim, we would kindly like to share us your professional experiences, knowledges, ideas, insights and practices story.

Participants bio-data

Age: ------------- gender: ------------specialty: ------------sector/place----------years of experience--------------------

1.From your perspective, at this time and future what are the main problems/barriers and challenges to the delivery quality of care, development and implementation of care management towards chronic disease/war injury in the Palestinian health care sector?

In other words, what are the factors do you think that your health system confronts, which may contribute to unsatisfactory performance or poor outcomes?

…………………………………………………………………………………………
…………………………………………………………………………………………
…………………………………………………………………………………………

2.What areas you are not strong in, as you would like to be? These might be areas you are working on improvements?

…………………………………………………………………………………………
…………………………………………………………………………………………
…………………………………………………………………………………………

3.What are the reasons, in your opinion, why health professionals do not use clinical practice guidelines? In other words, what are the main barriers for not using or adhering to use of guidelines for case management (chronic disease/war injury)?

…………………………………………………………………………………………………
…………………………………………………………………………………………………
…………………………………………………………………………………………………

4.What changes do you think would be required to enable health professionals to use a clinical guideline to effective management of chronic disease/war injury?

…………………………………………………………………………………………………
…………………………………………………………………………………………………
…………………………………………………………………………………………………

5.Frpm your perspective, what do you expect as the main barriers to shared-care of chronic disease /war injury among different health facilities in Palestine specially in Gaza Strip, for example; between PHC and hospitals, private and public sectors etc.…...?

…………………………………………………………………………………………………
…………………………………………………………………………………………………
…………………………………………………………………………………………………

6.According to the health service providers, we have for example these problems (please state major problems from healthcare providers), and according to patients we have these (xxxxxx) problems. As a decision maker, what do you plan or recommend for improving the system- or overcoming barriers?

…………………………………………………………………………………………………
…………………………………………………………………………………………………
…………………………………………………………………………………………………

7.From your perspective, how the current Palestinian political situation affects the care for chronic patients or war injured survivors? Please explain?

…………………………………………………………………………………………………
…………………………………………………………………………………………………
…………………………………………………………………………………………………

8-Is there anything else that you would like to add or to share with us?

…………………………………………………………………………………………………
…………………………………………………………………………………………………
…………………………………………………………………………………………………

The end of policy maker's interview
